# Infertility treatment for Chinese women with P450 oxidoreductase deficiency: Prospect on clinical management from IVF to FET

**DOI:** 10.3389/fendo.2022.1019696

**Published:** 2022-12-22

**Authors:** Yan Li, Cui-Lian Zhang, Shao-Di Zhang

**Affiliations:** Reproductive Medicine Center, People’s Hospital of Zhengzhou University, Henan Provincial People’s Hospital, Zhengzhou, Henan, China

**Keywords:** PORD, infertility, IVF, FET, HRT, pregnancy

## Abstract

Cytochrome P450 oxidoreductase deficiency (PORD) is a rare recessive disease with multiple clinical manifestations, which is usually diagnosed in neonates and children because of ambiguous genitalia or skeletal malformations. Moreover, the paucity of studies does not allow us to establish whether adult-onset PORD is associated with infertility. Here, we report clinical and laboratory findings in two phenotypically normal women diagnosed with PORD who underwent *in vitro* fertilization (IVF) and frozen embryo transfer (FET). We modified the gonadotropin stimulation protocol during controlled ovarian hyperstimulation (COH) and suggest the use of the vaginal 17β-estradiol route for endometrium preparation in hormone replacement therapy (HRT) cycles. We presume that PORD may be associated with infertility in several aspects, including disordered steroidogenesis, endometrium impairment, attenuation of drug metabolism, and the high risk of miscarriage. Our observations will help the early diagnosis and make a tailored approach to infertility management in adult-onset PORD.

## Introduction

Cytochrome P450 oxidoreductase (POR) serves as an electron donor to all microsomal cytochrome P450 (CYP) enzymes, including both drug-metabolizing and some steroidogenic enzymes (1, 2). PORD (OMIM: 613571 and OMIM: 201750) is a rare autosomal recessively inherited disease caused by homozygous or heterozygous mutations of POR, which mainly affect activities of P450c17 (17α-hydroxylase/17,20-lyase), P450c21 (21-hydroxylase), P450aro (aromatase), and lanosterol CYP 51 (14α-demethylase) (3, 4). Therefore, PORD is characterized by multiple clinical phenotypes depending on the severity of the causative POR mutations and the subsequent relative impairment of POR-dependent enzymes (5).

Currently, 119 patients have been reported with PORD worldwide, including 46 male and 73 female patients (6). The majority of PORD patients are diagnosed during infancy or childhood because of ambiguous genitalia or skeletal malformations (7, 8). Relatively few adults with PORD have been described (9, 10). Female patients with adult-onset PORD mainly exhibit primary amenorrhea, infertility, delayed pubertal development, or cystic ovaries. Patients are at risk of being misdiagnosed as suffering from 21-OHD or polycystic ovary syndrome (PCOS). The adrenal steroidogenesis was mainly affected in 21-OHD. PCOS is always accompanied by hyperandrogenemia, while PORD female adults mainly exhibit impaired sex hormone synthesis with combined 17α-hydroxylase and 21-hydroxylase deficiency (11). To date, only five women with PORD became pregnant after assisted reproductive techniques (ARTs) have been reported (10, 12–14). The *in vitro* fertilization (IVF) and frozen embryo transfer (FET) protocols have not been well summarized. Moreover, integrated approaches for infertility management and the potential complications of adult-onset PORD are not clear due to the limited number of patients.

Herein, we investigated two patients with a diagnosis of PORD who underwent ART therapy. We described their endocrine characteristics and specific pattern of response to controlled ovarian hyperstimulation (COH) based on clinical and laboratory findings. Moreover, the IVF and FET protocols leading to pregnancy were summarized. Furthermore, the relationship between PORD and infertility was hypothesized.

## Hypothesis

This report provides new insight into the relationship between infertility and adult-onset PORD, suggesting that infertility treatment of IVF and FET may improve the pregnancy outcomes in adult-onset PORD female patients.

### Clinical observations

In this study, two adult-onset PORD female patients presented with persistent high blood progesterone, low estradiol, and multiple ovarian cysts and received individual COH and FET protocols. Endocrine characteristics and pregnancy outcomes were observed.

### Case 1

A 35-year-old primary-infertile woman was enrolled in IVF treatment in February 2017. Menarche occurred at 14 years of age; thereafter, she had oligomenorrhea until hormone replacement therapy (HRT). She underwent cystectomy of multiple ovarian cysts three times when she was 15, 30, and 34, respectively. Her sister also has frequent ovarian cysts and received operations twice. Physical examination showed a height of 160 cm, a BMI of 24 kg/m^2^, a blood pressure of 114/65 mmHg, Tanner stage IV for breast development, Tanner stage II for axillary and pubic hair, normal genitalia, the absence of clinical hyperandrogenism, and no skeletal malformation. Adrenal cortical hyperplasia (CAH) remained undetectable on CT scans. Ultrasound showed a 6 × 5 cm cystic mass on the right ovary and a 3 × 2 cm cystic mass on the left ovary. Serum levels of basal estradiol (E2), dehydroepiandrosterone sulfate (DHEA-S), and testosterone (T) decreased while levels of follicle-stimulating hormone (FSH), progesterone (P4), and sex hormone-binding globulin (SHBG) increased (**Table 1**). Cortisol and adrenocorticotropic hormone (ACTH) levels as well as autoimmune antibodies were within the normal range (**Table 1**).

**Table 1:** Synopsis of the basal hormonal results.

	Patient 1	Patient 2	Normal range
Basal estradiol (pg/ml)	20.05	22.24	12.4–233
Basal progesterone (ng/ml)	1.14	7.93	0.2–1.5
Basal FSH (mIU/ml)	17.59	10.8	3.5–12.6
Basal LH (mIU/ml)	8.67	8.51	2.4–12.6
Basal total testosterone (ng/dl)	0.06	0.03	0.06–0.82
SHBG (nmol/l)	116.5	170.4	26.1–110
DHEA-S (μg/dl)	90.77	14.24	180–3910
TSH (mIU/ml)	1.41	4.68	0.27–4.2
TgAb (IU/ml)	18.90	8.00	0–115
Anti-TPOAb	19.01	100.26	0–34
ANA	Negative	Negative	
Antiβ2GP I antibody (U/ml)	2.6	24.18 first 26.48 second	0–18
ACA (MPL/ml)	0.9	11.92	0–12

Normal range: These gonadal hormone values represent normal values for female patients in the follicular phase; TgAb, thyroglobulin antibody; Anti-TPOAb, antithyroperoxidase antibody; ANA, antinuclear antibody; ACA, anti-cardiolipin antibody; Antiβ2 GP I antibody, antiβ2 glycoprotein I antibody, 1 month for the second time of re-examination.

She was identified with POR heterozygous variants NM_000941.2: c.1370G>A and NM_000941.2: c.1361T>C by whole-exome sequencing (WES) (**Table 2**). It is difficult to perform pedigree verification because her father died and her mother suffered from paralysis. Genetic testing results used GRCh37/hg19 as the human genome reference. The variant c.1370G>A (p.Arg457His) is predicted to be disease-causing. Though the variant c.1361.T>C (p.Leu454Pro) has not been reported in the population genetic database (gnomAD) or the pathogenic mutation database (Clinvar, HGMD), it was predicted to be deleterious and the damaging score is 0.91 (VarCards). Moreover, the region where the variation is located is highly conserved among different species. The variants identified by next-generation sequencing were subsequently validated using Sanger sequencing. Based on these data, the variant was classified as “Likely Pathogenic” according to the American College of Medical Genetics and Genomics (ACMG) guidelines.

**Table 2:** Summary of genetic molecular analyses.

	Patient 1		Patient 2	
	Mutation 1	Mutation 2	Mutation 1	Mutation 2
Transcripts	NM_000941	NM_000941	NM_000941	NM_000941
Nucleotide changes	c.1370G>A	c.1361T>C	c.1631T>C	c.1723G>A
Genotype	Het	Het	Het	Het
Inheritance	Not verified	Not verified	Father	Mother
ACMG	Pathogenic	possible	Possible	Possible
Disease	PORD	PORD	PORD	ABS

**Figure 1:**
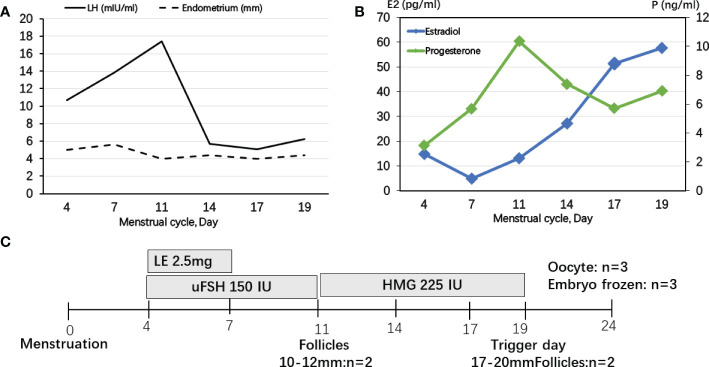
Controlled ovarian stimulation cycle in patient 1. **(A)** LH levels were slightly elevated to the maximum of 17.42 mIU/ml at day 11 of menstrual cycle, but decreased thereafter, but the endometrium was less than 6 mm during COH. **(B)** Serum E2 levels were only modestly increased and P levels were sustained above 5 ng/ml. **(C)** A protocol with letrozole (LE) and gonadotropins was used in patient 1. Ovulation was triggered by HCG on Day 19.

The IVF cycle was carried out on the fourth day of the menstrual cycle after oral contraceptive pretreatment for 1 month. The start dose was letrozole (LE) plus urine follicle-stimulating hormone (uFSH) 150 IU for 3 days and then uFSH 150 IU sustained for 4 days. Human menopausal gonadotropin (HMG) 300 IU was sustained for 8 days until human chorionic gonadotropin (HCG) trigger (**Figure 1C**). During FSH stimulation, serum E2 levels were only modestly increased and P4 levels were sustained above 5 ng/ml (**Figure 1B**). Luteinizing hormone (LH) levels were elevated to 17.42 mIU/ml on day 11 of the menstrual cycle, but decreased thereafter. The endometrium was less than 6 mm during COH (**Figure 1A**). It is noteworthy that E2 decreased for the first 3 days during COH because of letrozole (LE). A 10,000-IU dose of urine-derived HCG was administered to induce final follicular maturation, and oocytes were retrieved 36 h later. Three MII (Metaphase II) oocytes were picked up and three high-quality embryos with eight cells, even size, regular shape, and 5% fragmentation were cultivated 3 days later.

Hysteroscopy was conducted before FET. The hysteroscopic diagnosis of endometritis was based on the identification of diffuse hyperemia or hemorrhagic spots in the uterine cavity. After anti-inflammation therapy for 2 weeks and oral prednisone (5 mg/day) pretreatment, she underwent a subsequent FET cycle. A long-acting gonadotropin-releasing hormone (GnRH) agonist was used to suppress the pituitary–ovarian axis. She received oral estradiol valerate (Progynova, BAYER, Germany), 8  mg per day for 7 days, and endometrium thickness was 5  mm determined by transvaginal ultrasound (TVUS). The 2-mg daily dose of vaginal 17β-estradiol (Femoston, Abbott Biologicals, the Netherlands) was added for another 11 days. On the 18th day of HRT, when the endometrium thickness was 7.4 mm, oral dydrogesterone (Abbott Biologicals, the Netherlands) 40  mg daily was supplemented with Crinone (UK, Fleet Laboratories Ltd) *via* the vaginal route. After 3 days of progesterone administration, two embryos with eight cells were transferred on the fourth day. Pregnancy was diagnosed 14 days after embryo transfer by a β-HCG test, and a singleton pregnancy was confirmed 4 weeks later by transvaginal ultrasonography. She had high blood pressure just after 20 weeks of pregnancy and premature birth occurred after 36 weeks of pregnancy. A female baby was born by cesarean section in 2018 with no obvious abnormalities. The newborn length was 49 cm and the weight was 2.6 kg.

### Case 2

This primary-infertile 36-year-old patient visited our reproductive medical center in August 2020 after repeated attempts of IVF at other hospitals. She underwent two FET cycles and had an early pregnancy loss in the second cycle and then she was diagnosed as having PORD. No family history was found and the result of WES was NM_000941 c.1631T>C and NM_000941 c.1723G>A. The mutants were inherited from her father and mother, respectively, and classified as “Likely Pathogenic” according to the ACMG guidelines (**Table 2**).

She experienced primary amenorrhea and took dexamethasone for long-term treatment. Physical examination showed the following: a height of 165 cm, a BMI of 20.6 kg/m^2^, a blood pressure of 123/85 mmHg, Tanner stage V for breast development, difficulty in bending the metacarpophalangeal joints, narrow vagina and incomplete transverse vaginal septum, no clinical hyperandrogenism, and obvious skeletal malformation. Her first-visit ultrasound showed 2.6 × 2.2 cm, 2.0 × 1.9 cm, and 1.4 × 6.0 cm cystic masses on the right ovary and 2.7 × 2.4 cm, 1.7 × 1.3 cm, and 1.4 × 1.4 cm cystic masses on the left ovary. After HRT, a 1.9 × 1.2 cm cyst on the right ovary and a 1.4 × 0.9 cm cyst on the left ovary were found by the TVUS re-examination. No abnormality in the pituitary was found by magnetic resonance imaging (MRI) examination, as well as the adrenal CT scan. Cortisol levels were within the normal range. The serum level of anti-mullerian hormone (AMH) was 2.1 ng/ml. Serum levels of E2, DHEA-S, and T were reduced. Meanwhile, P4, SHBG, and TSH levels were increased. Moreover, her thyroid peroxidase auto-antibody (Anti-TPOAb), Antiβ2-GPI antibody, and anti-cardiolipin antibody (ACA) levels were increased (**Table 2**).

**Figure 2:**
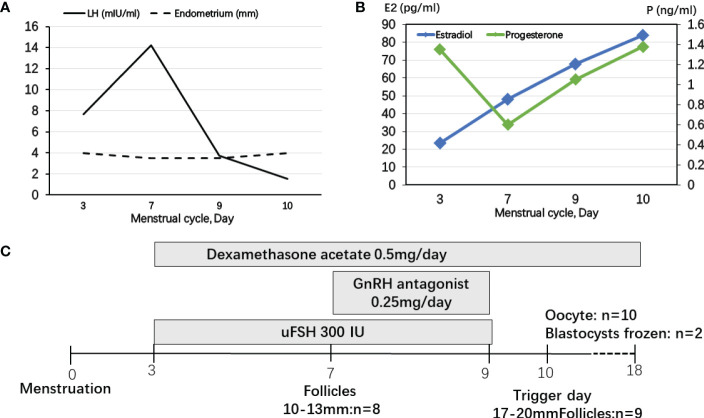
Controlled ovarian stimulation cycle in patient 2. **(A)** LH levels were elevated at the fourth day of FSH stimulating and decreased after using GnRH antagonist. The endometrium was less than 5 mm during COH. **(B)** During FSH stimulation, serum E2 levels were only modestly increased and P4 levels were controlled by dexamethasone. **(C)** The start dose was uFSH 300 IU for 4 days and then GnRH antagonist 0.25 mg was added until the day before the trigger day.

The IVF cycle was carried out on the fourth day of the menstrual cycle after oral contraceptive and dexamethasone pretreatment for 1 month. The start dose was urine-derived FSH 300 IU for 3 days and then GnRH antagonist 0.25 mg was added until the day before the trigger day (**Figure 2C**). During FSH stimulation, serum E2 levels were only modestly increased and P4 levels were controlled by dexamethasone (**Figure 2B**). LH levels were elevated on the fourth day of FSH stimulation. The endometrium was less than 5 mm during COH (**Figure 2A**). A 10,000-IU dose of urine-derived HCG was administered for final oocyte maturation. Oocyte aspiration was performed 35 h after the HCG trigger. The proportion of mature oocytes relative to the total number of oocytes aspirated was 90% (9/10). On day 6, two blastocysts evaluated as 3BB and 4BC using the Gardner system were frozen (15).

Hysteroscopy was conducted before FET. The hysteroscopic diagnosis of endometritis was based on the identification of diffuse hyperemia or hemorrhagic spots in the uterine cavity. After anti-inflammation therapy for 2 weeks, the HRT protocol was suggested for the patient. In the FET cycle, the vaginal and oral routes of 17β-estradiol (Femoston, Abbott Biologicals, the Netherlands) 8 mg per day were used for 20 days, and the endometrium finally reached 6.3 mm. FET under oral prednisone (5 mg/day) supplementation was performed. All of the day 6 blastocysts were transferred. Unfortunately, miscarriage occurred on the seventh week of pregnancy.

## Discussion

PORD is a complex disorder with many possible mutations affecting a large number of enzymes, and the most common mutations were R457H (25%) and A287P (24%) in 180 individual POR mutations from 90 patients (16). The present study provided two patients with complicated mutations in the POR gene and finally diagnosed as having PORD. Notably, primary amenorrhea/oligomenorrhea and infertility could be the main clinical manifestations when PORD female patients reached reproductive age after spontaneous puberty. Both patients were initially misdiagnosed as having 21-OHD or 17-OHD due to some overlapping features. Consistent with the five reported PORD female patients (14), our cases presented with impaired E2 and T production and elevated P4, whereas basal cortisol and ACTH remained within the normal range. Recurrent ovarian cysts also existed in our cases. Moreover, we found that the serum level of DHEA-S were low in both patients, accompanied by an elevated blood SHBG. Currently, the diagnostic criteria for adult-onset PORD have not been definitively established. PORD is the most complex of the various forms of CAH, because it affects the activity of several steroidogenic enzymes and yields a complex and variable pattern of abnormal steroid hormones. The 17,20-lyase activity of P450c17 is more sensitive to perturbations in electron transfer than its 17-hydroxylase activity; hence, the synthesis of DHEA, androstenedione, testosterone, and estradiol is more severely affected (17). Thus, adult women with PORD will have mildly elevated 17-OHP and low 19-carbon steroids. Some asymptomatic adult women with primary amenorrhea and cystic ovaries may seek infertility treatment (18, 19).

In previous studies, the GnRH agonist protocols were used in PORD female patients (10, 14, 20). P4 secreted by the corpus luteum strongly inhibited pulsatile GnRH and LH secretion (21). Progestin-primed ovarian stimulation (PPOS) is an ovarian stimulation protocol that can block the LH surge through oral progesterone instead of the traditional down-regulating or GnRH antagonist (22). In patient 1 without glucocorticoid pretreatment, we presumed that endogenous LH secretions could be suppressed in a PORD female patient with elevated P4, which is the “natural PPOS” protocol. P4 increased from cycle day 11 and no premature endogenous LH surge occurred thereafter (**Figure 1**). However, in patient 2, dexamethasone was used for pretreatment and P4 was decreased below 1 ng/ml. GnRH antagonist was used from cycle day 7 to prevent a premature endogenous LH surge (**Figure 2**). Thus, we recommend tailored COH strategies according to the P4 and LH variations during stimulation. In the present study, a 10,000-IU dose of urine-derived HCG was administered for final oocyte maturation. In both cases, the serum levels of E2 on trigger day were below 90 pg/ml due to PORD and clinicians freeze all embryos in fresh cycles because of elevated P4 levels during the follicular phase. In case 1, there were two follicles of 17–20 mm in diameter on the day of the HCG trigger (**Figure 1**). In case 2, there were nine follicles of 17–20 mm in diameter on the trigger day (**Figure 2**). Therefore, the probability of early- and late-onset ovarian hyperstimulation syndrome (OHSS) was decreased. Moreover, HCG trigger appears to improve the rate of oocyte recovery, and it is important for loosening the attachment of cumulus oophorus to the follicular wall so as to facilitate their recovery by follicle aspiration (23). There is uncertainty in the literature regarding both the optimal amount of time, “lag time”, from trigger administration to oocyte aspiration and whether an increased lag time translates into increased mature oocyte yields and better clinical outcomes (24–26). The ESHRE Working Group on Ultrasound in ART reports that intervals from 34 to 38 h are commonly used (27). In patient 1, a transvaginal ultrasound-guided oocyte aspiration was performed 34.5 h after the HCG trigger. In patient 2, oocyte aspiration was performed 35 h after the HCG trigger. The proportion of mature oocytes relative to the total number of oocytes aspirated was 100% (3/3) and 90% (9/10), respectively. Regarding the maturation rate, our data indicate that a 34- to 35-h lag time seems to be sufficient.

**Figure 3:**
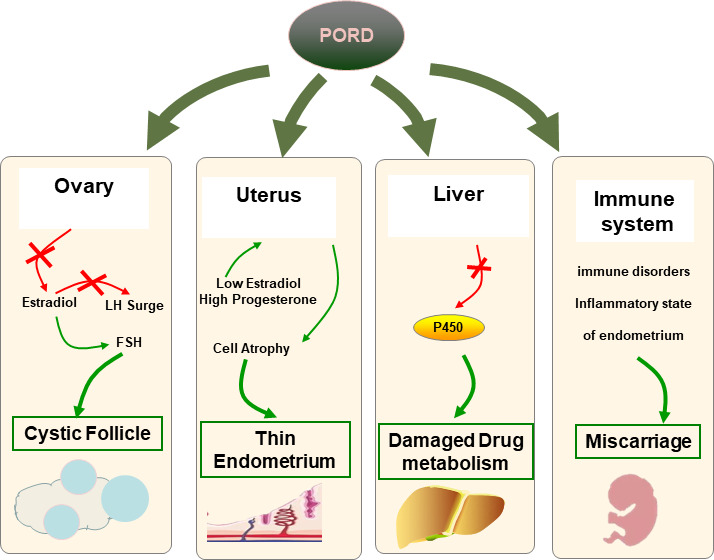
Hypothesis of associations with infertility in PORD. In ovaries, FSH fails to trigger the ovaries to produce enough E2 because of the impaired 17-α hydroxylase activities. Multiple cysts are caused by the dysfunction of the positive feedback loop to trigger the LH surge. The endometrium epithelial and stromal cells lack E2 stimulation and begin to atrophy. PORD may account for reduced hepatic drug metabolization. The risk of miscarriage may rise due to a dysfunctional endometrium and a disordered immune system.

Except for the recently reported five cases, no other studies focused on female infertility management in patients with PORD, especially for the HRT protocol. In the present study, hysteroscopy was performed before FET and vaginal administration of 17β-estradiol was recommended during HRT. Coincidentally, the results revealed endometritis in both cases. Recent literature suggests that endometritis is caused not only by local microbial infection but also by sex hormone imbalances (28, 29). Thus, the disordered ovarian sex steroids in PORD adults, especially E2 and P4, which are responsible for orchestrating the dynamic tissue remodeling in the uterus, were hypothesized by us to lead to the inflammatory state of endometrium (**Figure 3**). Additionally, PORD may account for reduced hepatic drug metabolization (30). In case 1, estradiol valerate was used orally for 7 days without obvious endometrium proliferation. Thus, we added the vaginal administration of 17β-estradiol. In case 2, we used 17β-estradiol in the whole HRT course according to previous experience. Estradiol valerate is a synthetic hormone that is extensively metabolized to estradiol in the liver. We propose that 17β-estradiol by vaginal administration would attenuate the hepatic first-pass effect and should be used for tailored infertility treatment in PORD (**Figure 3**). We suggest that hysteroscopy needs to be conducted before FET. The glucocorticoid treatment and sufficient luteal support may be beneficial in reducing the miscarriage rate.

In our cases, both women became pregnant after FET. However, one delivered a healthy baby, while the other experienced repeated early pregnancy loss with elevated autoimmune antibodies. As mentioned above, PORD is the most complex of the various forms of CAH. The miscarriage rate was significantly elevated in classic and non-classic CAH patients, but the reason remains unclear (31). PORD is associated with a decrease in E2 production and excessive ACTH stimulation. Emerging data suggest that estrogen deficiency can influence the immune system, including all levels of the innate (neutrophils, macrophages/monocytes, natural killer cells, and dendritic cells) and adaptive immune system (T and B cells) (32, 33). Also, the overactivation of the hypothalamic–pituitary–adrenal (HPA) axis may trigger the release of neurohormones, and subsequently, the activation of the HPA axis stimulates upregulation of stress hormones such as corticotropin-releasing hormone, ACTH, and glucocorticoids, which can consequently lead to altered inflammatory pathways and immune cell function affecting reproductive function (34). As reported, female patients respond more aggressively to self-antigens and are more susceptible to autoimmune diseases compared to their male counterparts (35). Thus, we propose that PORD may be associated with miscarriage in dysfunctional steroidogenesis and a disordered immune system (**Figure 3**).

In summary, considering the dysfunction of P450 enzymes, we made tailored COH strategies and modified the estradiol supplementation in PORD female patients. Furthermore, we suggest that hysteroscopy needs to be conducted before FET. The glucocorticoid pretreatment and sufficient luteal support may be beneficial to reducing miscarriage rate. Our findings may help the early diagnosis of late-onset PORD and expand the spectrum of appropriate infertility strategies. However, the findings of current research based on limited data need to be confirmed in more studies. The lack of patient data for side effect profiles for the long-term use of steroid supplementation is another limitation of the study. We hope that our findings will stimulate the research community to test our hypothesis further. In particular, it would be interesting to measure the immune response in women with PORD.

## Data availability statement

The original contributions presented in the study are included in the article/supplementary material. Further inquiries can be directed to the corresponding authors. Requests to access these datasets should be directed to 1162031398@qq.com.

## Ethics statement

Ethical approval was not provided for this study on human participants because Since this study used only de-identified patient data, no approval from our institutional review board was required. The patients/participants provided their written informed consent to participate in this study.

## Author contributions

YL contributed to the conception and design of the study and to the acquisition and interpretation of the data, prepared the first draft, and revised the manuscript. S-DZ and C-LZ contributed to the conception and design of the study. Each author listed on the manuscript has seen and approved the submission of this version of the manuscript and takes full responsibility for the manuscript.
